# Impedance Analysis of Capacitive and Faradaic Processes in the Pt/[Dema][TfO] Interface

**DOI:** 10.1021/acsami.3c15465

**Published:** 2024-01-22

**Authors:** Yingzhen Chen, Klaus Wippermann, Christian Rodenbücher, Yanpeng Suo, Carsten Korte

**Affiliations:** † Institute of Energy and Climate Research—Electrochemical Process Engineering (IEK-14), Forschungszentrum Jülich GmbH, 52425 Jülich, Germany; ‡ RWTH Aachen University, 52062 Aachen, Germany

**Keywords:** impedance analysis, electrolyte/electrode interface, ionic liquid, platinum electrode, oxygen reduction reaction

## Abstract

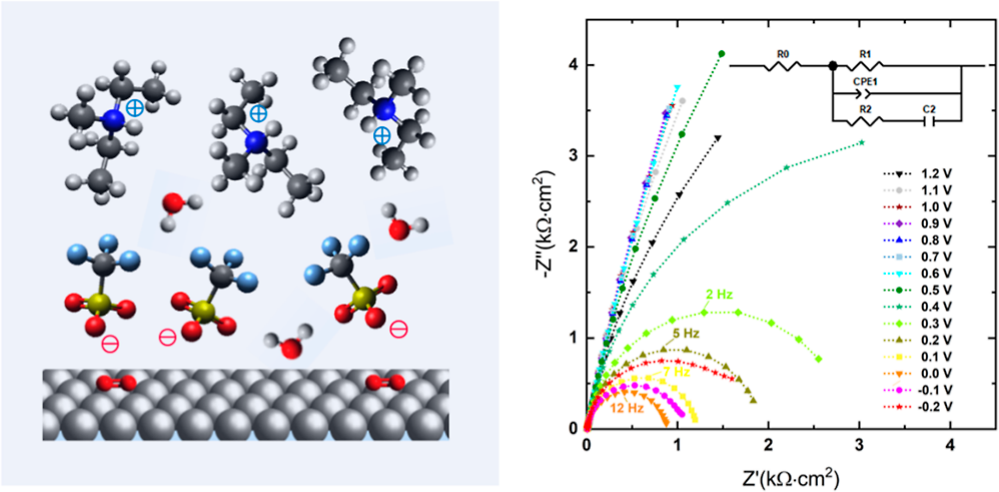

The electrochemical reaction kinetics, especially the oxygen reduction reaction (ORR) at the cathode, is crucial for the performance of a fuel cell. In this study, the electrochemical processes on a polycrystalline Pt electrode in the presence of protic ionic liquid (PIL) electrolyte diethylmethylammonium triflate [Dema][TfO] are investigated by means of cyclic voltammetry and electrochemical impedance spectroscopy. Since water is continually produced during fuel cell operation, the effect of the water content in the PIL has been intensively analyzed. In order to reveal the dependence of the interfacial reaction characteristics on the electrode potential, the impedance spectra were simulated by an equivalent circuit whose parameters can be related to both Faradaic and capacitive processes. Two interfacial resistances were identified, which differ by about 3 orders of magnitude. The larger one is a charge transfer resistance that can be associated with slow Faradaic processes like the ORR and platinum oxidation/oxide reduction. The smaller resistance is probably linked with fast processes that involve water molecules, such as hydrogen deposition and oxidation. The high- and midfrequency capacitive processes are attributed to “classical” double layer and pseudocapacitive behavior, similar to those identified under nitrogen atmosphere.

## 1 Introduction

Ionic liquids are known and used as polar solvents in e.g., organic synthesis due to negligible vapor pressure, high thermal stability, and nonflammability. Another growing field of application is as novel electrolytes in electrochemical devices due to their wide electrochemical windows and good ionic conductivity. Recently, protic ionic liquids (PILs) immobilized in a host polymer have been considered as potential electrolytes for proton exchange membrane fuel cells (PEMFCs) to enable atmospheric operation above 100 °C.^1−4^ A prerequisite for the technical application of a PIL electrolyte in fuel cells is a detailed understanding of the electrochemical kinetics of fuel-cell-relevant electrode processes, like the oxygen reduction reaction (ORR) at the cathode and the hydrogen oxidation reaction (HOR) at the anode. Since the kinetics of the ORR is much slower than the HOR, the performance of a PEMFC relies primarily on the ORR, which is the limiting factor for the current density in a PEMFC. Thus, the interfacial reaction mechanism and kinetics and the structure and properties of the double layer, where the ORR takes place, are crucial for the further optimization of the ionic structure and materials properties of a PIL electrolyte.

The structure of the electrical double layer in an ionic liquid in the vicinity of the electrode surface is more complex compared to a “classical” aqueous electrolyte.^5,6^ In an aqueous solution, an excess of a dielectric solvent is usually present that consists of neutral (water) molecules. The ions, which act as mobile charge carriers, are solvated (solvate shell), and the ionic charges are partially shielded by the dielectric interactions. An IL consists of only charged cations and anions (“salt melt”). Owing to the high density of charge species in ionic liquids, strong intermolecular interactions, i.e., Coulomb forces, hydrogen bonding, van der Waals forces, and steric effects, play a significant role in the local arrangement of the ions, ordering phenomena in the nanoscale (superstructures) and their response to electrical polarization.^5^ The Gouy–Chapman–Stern double layer model based on the Poisson–Boltzmann equation is assuming a continuous dielectric medium, i.e., a low concentration of ions far separated from each other, and is therefore not suitable for describing the ionic liquid/electrode interface. In a neat ionic liquid, alternating layers of densely packed anions and cations can be found close to a charged electrode, according to molecular dynamics simulations and atomic force spectroscopy.^7−10^ Taking the finite volume of the ions into account, Kornyshev presented a mean-field lattice gas model. Depending on the relative ion density, a “bell-shaped” or “camel-shaped” course of the double-layer capacitance as a function of the electrode potential is suggested.^11−13^ These features have been experimentally verified by means of electrochemical impedance spectroscopy.^14,15^ Moreover, two capacitive processes at the interface were found that occur on a time scale of milliseconds and seconds, respectively.^16−18^

Most investigations focus on capacitive processes in neat aprotic ionic liquids under nitrogen atmospheres. Despite the progress in understanding the structure of the electrified interface between an electrode and an ionic liquid and its capacitive behavior,^5,19,20^ the impact of interface on the Faradaic processes remains unclear. In a fuel cell, the Faradaic processes, especially the ORR, predominate. In this study, we aim to investigate the capacitive and Faradaic processes at a PIL/Pt interface by means of cyclic voltammetry and electrochemical impedance spectroscopy. In order to investigate the cathodic interface reactions on a fundamental level, a half-cell geometry employing a polycrystalline Pt electrode in an oxygen-saturated liquid PIL electrolyte was used as a model system. Oxygen was dissolved in the PIL in order to imitate the gas phase at the fuel cell cathode. The PIL diethylmethylammonium triflate [Dema][TfO] was selected as a model electrolyte, as it has been considered the most promising candidate for PEMFC applications owing to good performance in studies testing its performance at high temperatures.^1^ For a quantitative impedance analysis, an equivalent circuit was proposed by considering both physical and statistical aspects. The interfacial characteristics, such as electrolyte resistance, the charge transfer resistance, and capacitances, have been studied as a function of the electrode potential. As water is unavoidable in fuel cell operation and has a strong impact on the interfacial structure and ORR activity,^20−22^ the influence of various amounts of residual water has also been investigated.

## 2 Experimental Section

[Dema][TfO] was used as received (CAS no. 945715-39-9, IoLiTec-Ionic Liquids Technologies GmbH, Germany). The sample was analyzed by nuclear magnetic resonance spectroscopy, which shows that the purity of the [Dema][TfO] is more than 98%. The impurities of halides determined by ion chromatography are less than 500 ppm. The samples were prepared by mixing the PIL and purified water (Milli-Q, Merck KGaA) with a molar ratio of 2:1, 1:1, 1:2, 1:3, and 1:4, corresponding to 37, 54, 68, 76, and 80 mol % of water. The [Dema][TfO] without additional water is referred to as a “neat” ionic liquid. Detailed information about the water content before and after the EIS measurements was determined *via* Karl–Fischer titration, with the results shown in Figure S1. Owing to the hygroscopicity of [Dema][TfO], the water content of the neat sample was found to increase from 0.24 ± 0.01 to 2.64 ± 0.08 wt %, namely, from 3.07 ± 0.04 to 26 ± 1 mol % after the measurement. The water content of other samples was almost constant before and after being measured.

The electrochemical measurements were carried out in a cell with a three-electrode configuration within a Faraday cage (VistaShield, Gamry Instruments, USA) using a potentiostat (Zennium, ZAHNER Elektrik GmbH, Germany), as reported in a previous study.^23^ The cell was constructed from a cylindrical platinum vessel, which also served as the counter electrode. A platinum wire with a diameter of 1 mm placed in the center of the vessel was used as the working electrode (99.95%, Goodfellow GmbH, Germany). A hydrogen-saturated palladium wire served as the reference electrode (99.95%, Goodfellow GmbH, Germany). In an aqueous acidic electrolyte, the potential of Pd–H is 50 mV *vs* RHE at 25 °C.^23^ In the mixture of [Dema][TfO] and water, the reference potential is 18, 29, 37, 43, 46, and 47 mV *vs* RHE for the PIL with 2.6, 37, 54, 68, 76, and 80 mol % of water, respectively. Oxygen saturation was achieved by purging the ionic liquid with an oxygen flow rate of 10 mL/min for 1 h and then keeping the electrolyte under an oxygen atmosphere during the measurements. Cyclic voltammograms (CVs) were run 10 times with a scan rate of 100 mV/s between −0.2 and 1.2 V. The last cycle is shown and discussed for the analysis. A series of impedance measurements were performed with a 20 mV AC amplitude in a frequency range from 100 kHz to 1 Hz. The electrode potential sequentially decreased in steps of 0.1 V from 1.2 to −0.2 V. Before recording a spectrum, the potential was kept for 30 s in order to attain stationary conditions. The experimental data were fitted by means of Z-View software (version 2, Scribner Associates Inc., USA) using the equivalent circuit model, shown below.

## 3 Results and Discussion

### 3.1 Electrochemical Characterization of the IL/Water Mixture

**Figure 1:**
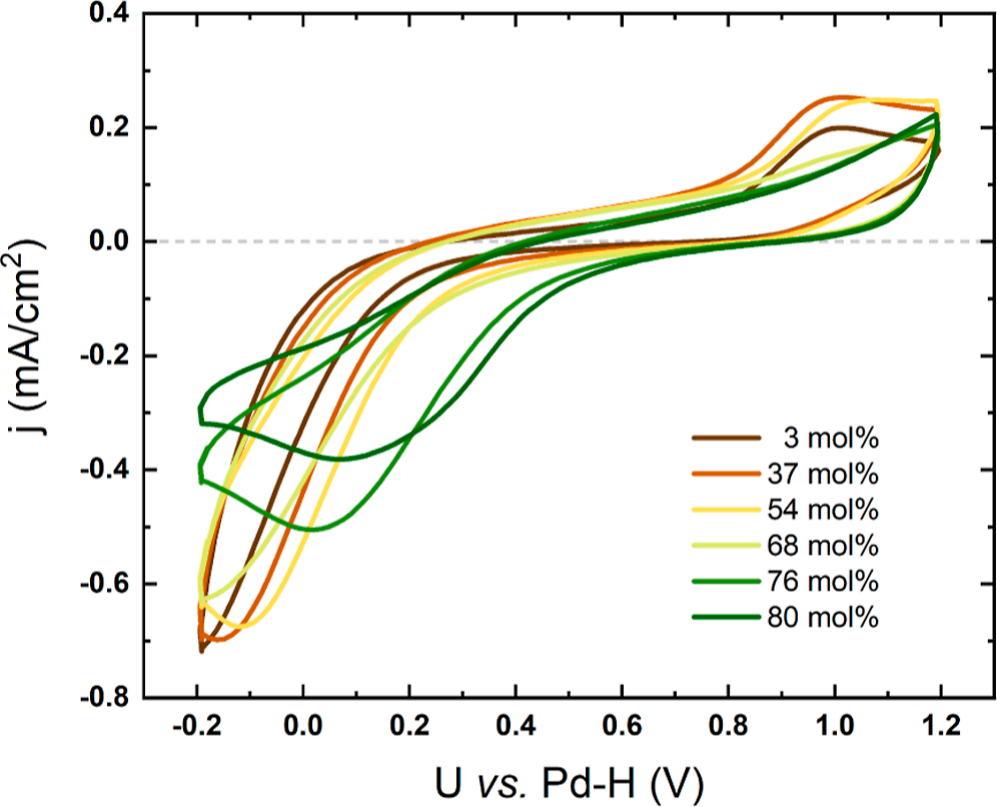
Cyclic voltammetry of [Dema][TfO] with various water contents under an O_2_ at a scan rate of 100 mV/s.

The characteristic CV curves of a mixture of [Dema][TfO] and H_2_O recorded in a potential range from −0.2 and 1.2 V are shown in Figure 1. The open circuit voltage (OCV) of all investigated samples was about 0.8 V *vs* the Pd–H reference electrode, where the current density was close to zero during the cathodic scan. The reduction wave of neat [Dema][TfO] starts around 0.3 V, which corresponds to the ORR at the Pt electrode and the reduction of oxide species at the Pt electrode surface. As the water content increases, the onset potentials of both processes shift to higher values. This indicates that the addition of water decreases the activation energy of the reduction reactions. The anodic peaks observed at around 1 V are indicative of oxide formation on the platinum surface.

### 3.2 Impedance Characterization of the PIL/Water Mixture under O_2_

#### 3.2.1 Complex Plane Plots of EIS Measurements

**Figure 2:**
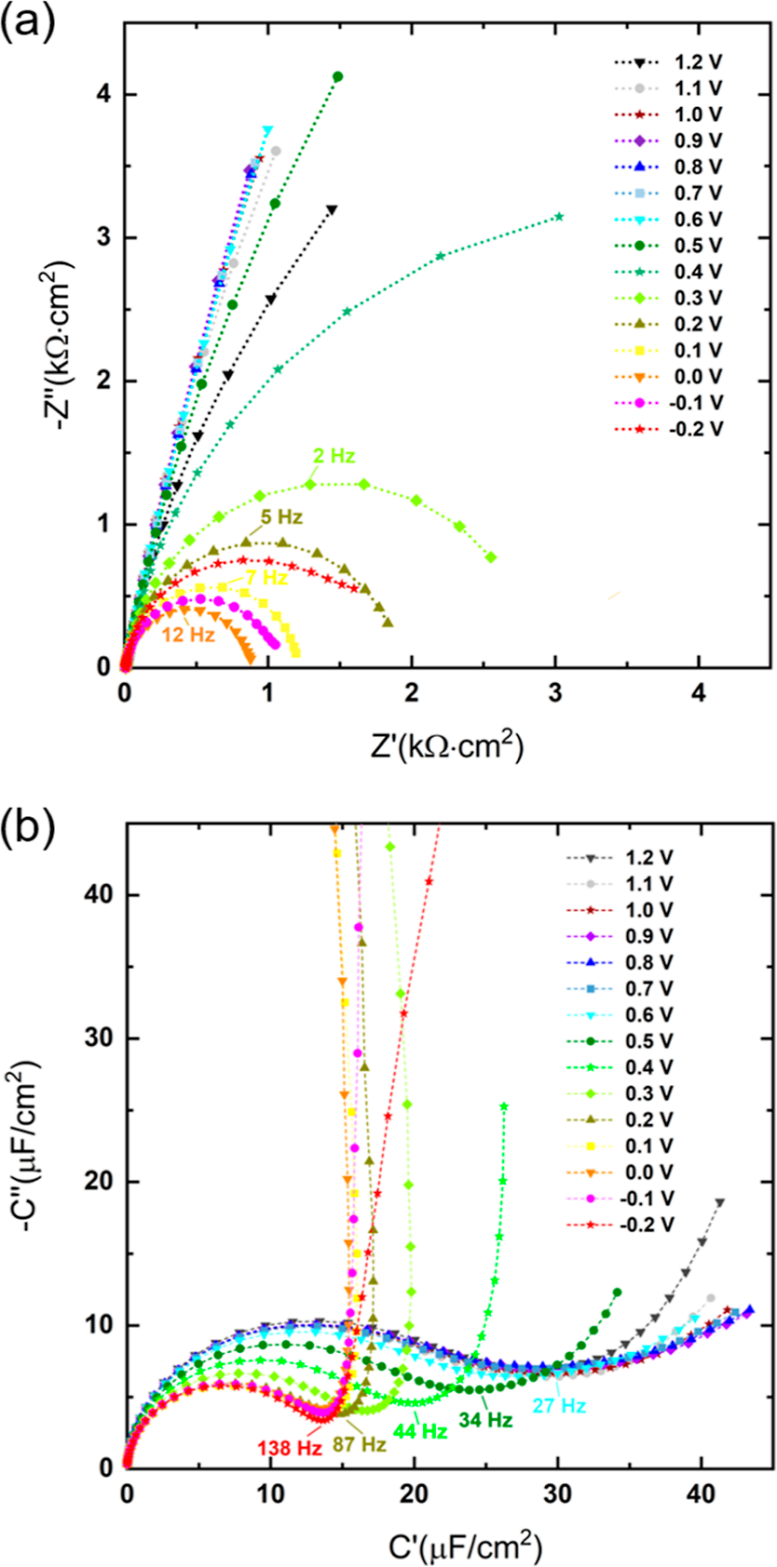
Impedance spectrum of [Dema][TfO] with 50 mol % water at a Pt electrode under an O_2_ atmosphere: (a) Nyquist plot and (b) complex capacitive plane plot. The voltage values indicate the potential applied to the Pt working electrode against the Pd–H reference electrode. The lines are a guide to the eyes.

Figure 2a illustrates the impedance response of [Dema][TfO] with 54 mol % of water represented in a complex impedance plane (Nyquist plot), where *Z*′ and *Z*″ are depicted as the real and imaginary components of impedance *Ẑ*, respectively. The impedance data are normalized by multiplication with the electrode surface area of 0.26 cm^2^. Each measured point corresponds to a different frequency in the range of 100 kHz to 1 Hz. The characteristic frequencies, indicated by the apexes of the semicircles, i.e., the maxima of the imaginary parts, correspond to the time constants of the underlying electrochemical processes. A semicircle is observed at a potential of 0.0 V with a characteristic frequency of 12 Hz. With increasing cell potential of up to 0.7 V, the diameter of the semicircle increases and the characteristic apex frequency decreases. A complete semicircle cannot be observed due to the limited measuring points in the low-frequency range. In the potential region between 0.8 and 1.1 V, high phase angles >80° (Figure S2) reveal the highly capacitive nature of the processes in this potential region. However, the presence of concurrent Faradaic processes, i.e., the redox reactions of residual water and the subsequent Pt-oxidation, leads to a tilted line in this potential region that deviates from ideal capacitive behavior, which could result in a straight vertical line.

Plotting the data in the complex capacitance plane (CCP), which represents the imaginary *C*″ *vs* the real part *C*′ of the complex capacitance *Ĉ*, was found to be more suitable than the representation in a Nyquist plot. The impedance data were converted into CCPs *via Ĉ* = 1/(*j*ω*Ẑ*). The obtained values were normalized by division with the electrode surface area and are plotted in Figure 2b. For cell potentials above 0.6 V, an asymmetrical semicircular feature with a linear tail is observed, whereas only tilted lines are visible in the Nyquist plot in Figure 2a. The frequencies that correspond to the transition between the semicircle and the vertical line are marked in Figure 2b. The “transition frequency” increases with decreasing potential, i.e., with increasing Faradaic current. This means that capacitive processes are increasingly masked by Faradaic reactions such as ORR.

A depressed asymmetrical semiarc provides a clear indication that more than one time constant is required to describe the process. A similar feature was observed by the Roling group when studying the interfacial behavior of aprotic ionic liquids at a gold electrode.^16−18^ They found two depressed semicircles at high and intermediate frequencies, followed by a tilted line at low ones. At a cell potential of 0.7 V and lower, the semiarc becomes small and the line tends to be more parallel to the *y*-axis; see Figure 2b. A nearly vertical line can be observed if the cell potential is below 0.4 V. This is caused by highly resistive behavior and indicates a dominating Faradaic process. The latter corresponds to the appearance of a semiarc in the Nyquist plot in this potential range.

#### 3.2.2 Modeling Considerations

For a quantitative analysis, impedance results are commonly fitted to an appropriate model, which should result in a good fit to the measured spectra and hence be able to describe the electrochemical processes of the systems, i.e., Faradaic reactions at the interface and mass/ion transport in the bulk. One of the common approaches is fitting the impedance results to an equivalent electric circuit model. A fit of the spectrum is made to identify the contribution of the single components present in the equivalent circuit.

**Figure 3:**
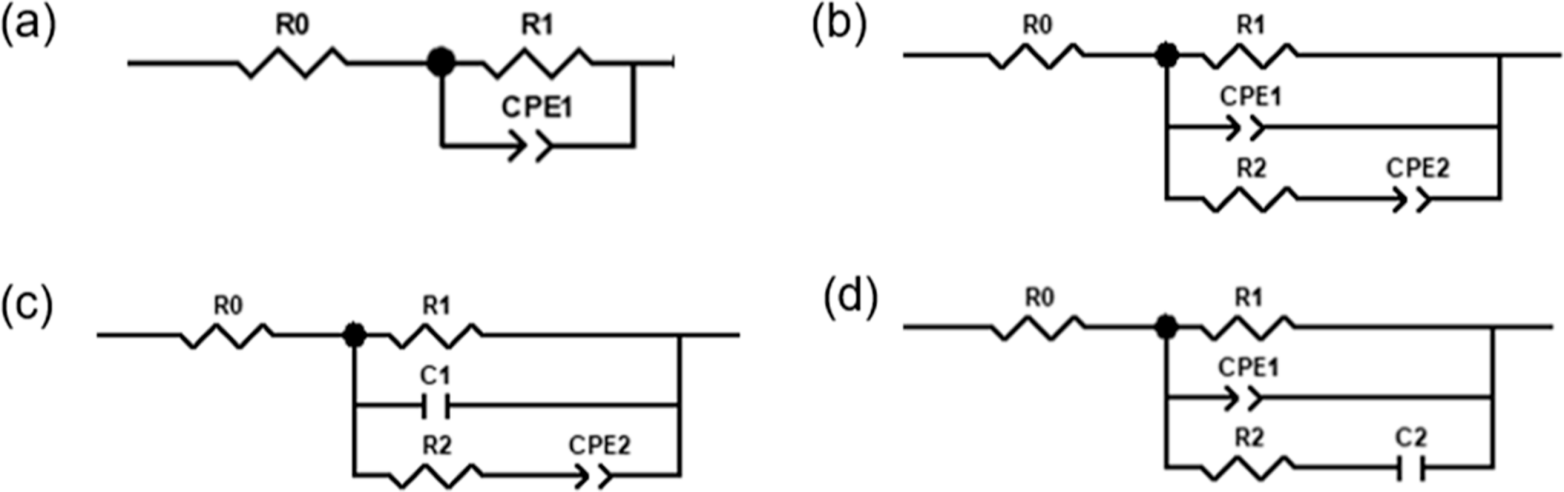
Discussed equivalent circuits are (a) Randles circuit model and (b–d) the modified models suggested in this study.

One of the typical electrochemical models is the Randles circuit, which consists of a parallel combination of a resistance *R*_1_ and constant phase element (CPE_1_), in series with the electrolyte resistance *R*_0_; see Figure 3a. *R*_1_ relates to the charge transfer process at the electrode interface, whereas CPE_1_ models the electric double layer capacitance. The impedance of a CPE is given by *Ẑ* = *Q*^–1^(*i*ω)^−α^, where *Q* and α are the characteristic parameter of the CPE. The exponent α describes the phase shift and varies between 0 and 1. If α = 1, the constant phase element is identical to a capacitor with a capacitance equal to *Q*. Because α < 1, the constant phase element CPE_1_ is used instead of a capacitor. The nonideal capacitive behavior can be associated with either the roughness of the electrode^24−26^ or slow processes that occur at the electrode,^27,28^ e.g., specific ion adsorption on the electrode or reorientation of ions. Although the Randles circuit model is able to provide a statistically adequate fit to measure the EIS data (see Figure S4), this model could oversimplify the description of the multiple processes involved at ionic liquid/electrode interfaces. The existence of more than one time constant is suggested from a depressed asymmetrical semiarc in the complex capacitance plot in Figure 2b. This cannot be reflected by only one CPE representing all capacitive processes. Therefore, a more detailed model must be constructed.

In order to resolve the capacitive processes with different time constants at the ionic liquid–solid interface, the complex capacitance spectra were fitted to an empirical Cole–Cole type expression , similar to that published by Drüschler *et al.*(17) The nonideal behavior of each capacitive process is represented by three parameters: a differential capacitance *C*_*i*_, an exponent α_*i*_ and a time constant τ_*i*_. Drüschler *et al.* used this model to investigate the double-layer capacitance of aprotic ionic liquids at a gold electrode under a nitrogen atmosphere. Because the complex capacitance is given as the sum of capacitances, *C*_*i*_, these must be connected in parallel. A fast capacitive process (>20 Hz) is attributed to the charging of the double-layer, whereas a slower one (<20 Hz) to a restructuration process of the electrode surface or of the ions in the innermost layer. This model is well-suited to describing electrified interfaces, in which capacitive processes dominate over a large range of frequencies. This is surely the case for aprotic ionic liquids at a gold electrode under nitrogen atmosphere, where slow Faradaic processes generate a tilted line at low frequencies. Such a resistive behavior has been reported by Drüschler *et al.*(17) and can also be found in our own previous study on the electrode kinetics of PIL electrolytes purged with nitrogen.^20,22^ However, as soon as the resistance and time constant of a Faradaic process become smaller, the resistive line appears at higher frequencies and will mask large parts of the CCP plot. This is, for example, the case (see Figure S3), if the PIL electrolyte is purged with oxygen instead of nitrogen, allowing the ORR to take place. Consequently, the attempt to fit our EIS data to the Cole–Cole type expression will result in large errors of the fitting parameters, especially for the masked midfrequency capacitance. As can be seen in Figure S5, the average error of *C*_2_ is around 47%, which is much higher than that in the high-frequency range with 19% error of *C*_1_. This reveals the limitation of this model when Faradaic processes predominate.

Therefore, a modified model to characterize the PIL/electrode interface is required that represents both capacitive and Faradaic processes. We modified the common Randles circuit model with additional capacitance. This additional capacitance should take into account that at least two capacitive processes can be observed in ionic liquid electrolytes and represents the (more or less masked) midfrequency capacitance in the CCP plots. Analogous to the parallel connection of capacitive processes that was used for the former analyses with the Cole–Cole equation, the additional capacitance was connected parallel to the first one. Initially, two CPE elements were used instead of the capacitors, taking into account the nonideal capacitance behavior. In order to distinguish the two CPE elements in the circuit, a further resistor *R*_2_ was added in series with the second CPE element. This new model is depicted in Figure 3b. CPE_1_ accounts for the capacitive processes at high frequencies, whereas CPE_2_ represents those at lower frequencies.

**Figure 4:**
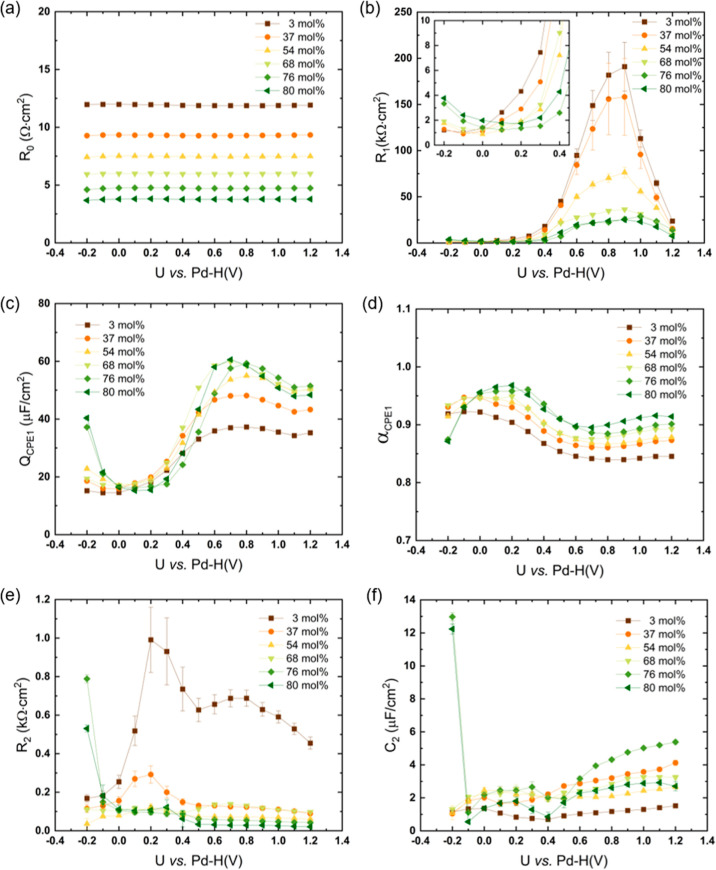
Fitted equivalent circuit parameters (a) *R*_0_; (b) *R*_1_; (c) *Q*_CPE1_; (d) α_CPE1_; (e) *R*_2_; and (f) *C*_2_ as a function of potential using the model with CPE_1_ and *C*_2_, as shown in Figure 3d. The lines are a guide to the eyes.

Fitting the data using this model leads to large errors in the parameters (Figure S6 and Table 1). In order to avoid overparameterizing, either the CPE_1_ or CPE_2_ element was substituted by an ideal capacitance *C*. The fitting results of a *C*_1_–CPE_2_-based model according to the equivalent circuit in Figure 3c are depicted in Figure S7, whereas the fitting results of a CPE_1_–*C*_2_-based model corresponding to Figure 3d and are shown in Figure 4. As depicted in Table 1, the overall error of the CPE_1_–*C*_2_-based model significantly decreases; the goodness of fit (see the sum-squared and chi-squared parameter χ^2^) from the CPE_1_–*C*_2_-based model is not significantly different compared to the CPE_1_–CPE_2_-based one. Although the fitting errors of *R*_2_ and *T*_2_ are diminished in the *C*_1_–CPE_2_-based model, the error of *R*_1_ becomes over 4 times larger, and the goodness of fit is lower compared to the other two models. In summary, considering both the overall and individual fitting errors, the CPE_1_–*C*_2_-based model shown in Figure 3d appears to be the most appropriate one.

**Table 1:** Fitting Error of the Equivalent Circuits

model	chi-squared	sum-squared	*R*_0_ (%)	*R*_1_ (%)	*R*_2_ (%)	*C*_1_/*Q*_CPE1_ (%)	*C*_2_/*Q*_CPE2_ (%)	α_CPE1_ (%)	α_CPE2_ (%)
Randles model	4.77 × 10^–4^	0.045	0.52	22.80		1.14		0.19	
CPE_1_–CPE_2_ model	1.34 × 10^–4^	0.012	0.38	2.65	56.96	17.24	73.53	14.16	7.15
*C*_1_–CPE_2_ model	2.08 × 10^–4^	0.019	0.37	10.68	9.19	1.68	2.49		0.50
CPE_1_–*C*_2_ model	1.45 × 10^–4^	0.013	0.30	3.63	13.04	1.37	7.63	0.17	

#### 3.2.3 Analysis of Fitting Results

The following analysis is based on the equivalent circuit model in Figure 3d. The fitted parameters and fitting errors are plotted against the applied cell potential; see Figure 4. All obtained parameters were normalized to the real surface electrode area of 0.26 cm^2^.

The Ohmic resistance *R*_0_ accounts for the PIL electrolyte resistance. As shown in Figure 4a, *R*_0_ is almost independent of the electrode potential. The value of *R*_0_ for neat [Dema][TfO] is about 11.92 ± 0.04 Ω·cm^2^. A decrease in electrolyte resistance is found with increasing water content in the PIL. The electrolyte resistance decreases from 7.48 ± 0.03 to 5.97 ± 0.02, 4.73 ± 0.04, and 3.77 ± 0.03 cm^2^ for mixtures of IL and water at molar ratios of 1:1, 1:2, 1:3, and 1:4, respectively. The addition of water results in a decreasing viscosity of the bulk PIL and therefore, because of the strong coupling of viscosity and conductivity, in an increase in the specific conductivity.

*R*_1_ corresponds to kinetic resistance, which is associated with redox reactions at the electrode/electrolyte interface. As depicted in Figure 4b, the maximum value of *R*_1_ for neat [Dema][TfO] can be observed near a potential of 0.9 V. This potential value is close to the open circuit voltage (OCV), which coincides with the highest slope of the *j*/*U* curve in the CV, as depicted in Figure 1. If the cell potential increases from 0.9 to 1.2 V, the kinetic resistance *R*_1_ descends significantly from 191.0 ± 26.2 kΩ·cm^2^ to a value of 23.7 ± 0.6 kΩ·cm^2^. This can be attributed to the adsorption of residual water and subsequent oxide formation on the platinum surface. With increasing overpotential, the oxidation reaction rate increases, and the kinetic resistance *R*_1_ decreases, correspondingly. In the potential range from 0.9 to 0.3 V, the high *R*_1_ values of neat [Dema][TfO] correlate with the flat part of the *j*/*U* curve in the CV; see Figure 1. The onset potential of the ORR is found at about 0.2 V. As the potential goes from 0.2 to −0.2 V, the kinetic resistance of neat [Dema][TfO] drops from 4.32 ± 0.04 to 1.200 ± 0.004 kΩ·cm^2^. The ORR performance is dominated by the electron and proton transfer to the adsorbed O_2_ molecule on the Pt electrode.^4^ In the case of the neat PIL, the protic cation serves as a proton donor in the rate-determining step. If water is added, the protolysis equilibrium BH^+^ + H_2_O ⇌ B + H_3_O^+^ is established and results in the formation of H_3_O^+^, which acts as the dominating proton donor in the ORR kinetics. It can be seen in Figure 4b that the kinetic resistance *R*_1_ decreases with increasing water content in the PIL. This is in accordance with the observed shift in the onset potential of the ORR by 0.6 V to positive values when the water content increases up to 80 mol %. At higher water contents (>50 mol %), the kinetic resistance *R*_1_ increases at a high overpotential (*U* < 0.2 V), as depicted in the inset of Figure 4b. This could be related to the hydrogen evolution reaction and the production of hydrogen gas bubbles, blocking active sites on the catalyst surface.

The presence of two capacitive processes occurring on different time scales has been accounted for in the modeling of the interfacial processes of [Dema][TfO] at the platinum electrodes. As is shown in Figure 4c,d,f, the larger capacitance, which is represented by a constant phase element, is indicated by *Q*_CPE1_ with its exponent α, whereas the smaller one is represented by *C*_2_, respectively. The parameter α describes the degree of nonideality for the capacitance. It can be seen in Figure 4d that there is a general trend of the CPE exponent approaching unity as the water content increases, suggesting that it becomes an ideal capacitor with the addition of water. This could result from hydrated ions that are formed with increasing water content, which leads to a decrease in ion adsorption on the electrode surface and an increase in the homogeneity of the charge distribution in the electrode/electrolyte interface.

**Figure 5:**
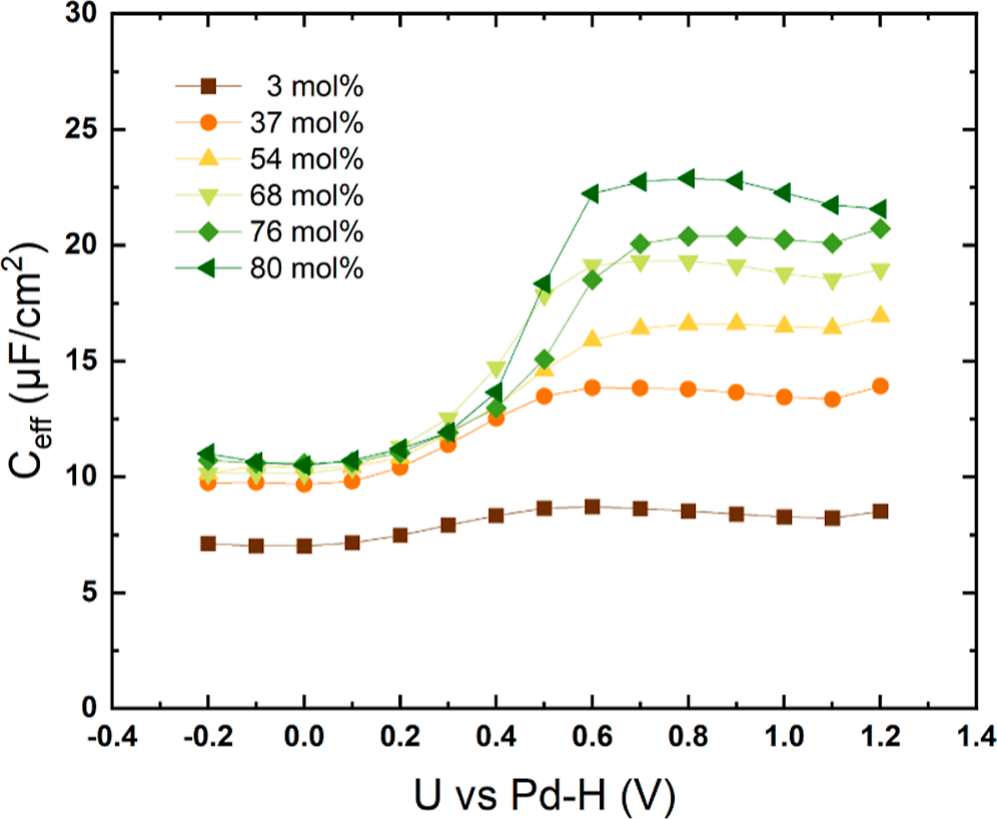
Effective capacitance is associated with the high-frequency capacitive process in the double layer.

The capacitance of this capacitive process cannot be represented by *Q*_CPE1_, as the exponent α is smaller than 1. In order to estimate the effective capacitance (*C*_eff_) from these CPE parameters, the following formula was used (see Hirschorn *et al.*(29))
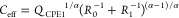
where *Q* is the CPE time constant; α is the CPE exponent; *R*_0_ is the Ohmic resistance and *R*_1_ is the kinetic charge-transfer resistance. As is shown in Figure 4, *R*_1_ is 2–4 orders of magnitude larger than *R*_0_. In this case, an effective capacitance can be calculated using the following, simplified equation that is equivalent to Brug’s formula^27^
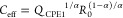
Note that Brug derived this equation for a simple Randles circuit. Because our equivalent circuit is more complex, the effective capacitances calculated from the formula described above are only approximate values. The obtained effective capacitances are depicted in Figure 5. Water is found to have a strong effect on the capacitive process. The potential dependent capacitance *C*_eff_ is increasing with higher water content especially in the potential range above 0.6 V (see Figure 5). Water molecules can replace the ions of the ionic liquid and accumulate at the charged electrode surface.^21,30^ At high potentials (>0.6 V), absorbed water reacts with Pt and forms oxide species, which implies a charge flow that leads to an increase in *C*_eff_ due to pseudocapacitances. This effect becomes more prominent at higher water concentrations. As is shown in Figure 5, neat [Dema][TfO] exhibits only a slight increase in *C*_eff_ in the potential range from 0.1 to 0.6 V. However, a steeper increase of *C*_eff_ from 0.3 to 0.6 V is observed with higher amounts of water. When the water concentration is increased from 37 to 80 mol %, *C*_eff_ increases from 13.8 to 22.9 μF/cm^2^ at 0.8 V. Furthermore, the increase in the capacitance can also result from a higher average permittivity of the sample with additional water, as water has a higher permittivity of 78.5 at 25 °C compared with ones in ionic liquids. Apart from OH-functionalized ILs, the permittivity of (protic) ionic liquids is usually not higher than 30–40.^31^

The branch in the equivalent circuit in Figure 3d with *R*_2_ and *C*_2_ (see also Figure 4e,f) represents a second interfacial process, whose nature is yet to be determined. This does not, however, mean that *R*_2_ is exclusively linked to *C*_2_ and *vice versa*. It should be kept in mind that because of the complex (parallel/serial) connection of the resistances and capacitances, an unambiguous correlation of a resistance with a capacitance in the equivalent circuits shown in Figure 3b–d is not possible. Because *R*_2_ is about 3 orders of magnitude smaller than *R*_1_, the associated process must be much faster than that of *R*_1_. Conversely, *C*_2_ represents a slower capacitive process compared to the “classical” double layer charging based on ion rearrangement that is associated with the effective capacitance *C*_1._ This is clearly evident from a comparison of the capacitances in Figures 4f, 5, and S5a,b, where the effective capacitance *C*_1_ in Figure 5 is similar to the high-frequency capacitance shown in Figure S5a and the smaller capacitance *C*_2_ in Figure 4f corresponds to the midfrequency capacitance in Figure S5b. It is probable that the capacitive process associated with *C*_2_ takes place in the innermost layer, such as the adsorption/desorption of ions at the electrode surface.^17^ As is shown in Figure 4f, *C*_2_ is smaller than 6 μF/cm^2^, except for two points probably related to the hydrogen evolution reaction at −0.2 V for high water contents of 76 and 80 mol %.

The maximum resistance *R*_2_ appears at about 0.2 V *vs* Pd–H for PILs with a low water content (<50 mol %). The recalculated values *vs* RHE of ≈0.22–0.24 V (see the Experimental Section) are close to the potential of zero charge (PZC) of neat [Dema][TfO], i.e., 271 ± 9 mV *vs* RHE.^32^ The molecular dynamics simulations of Feng *et al.* have shown that the water coverage exhibits a minimum close to the PZC.^21^ If *R*_2_ is associated with processes that involve water, this might explain the maximum of *R*_2_ being close to the PZC. This is supported by the dependence of *R*_2_ on the water concentration: whereas *C*_2_ exhibits no clear trend with respect to the water content, *R*_2_ increases significantly at low water contents smaller than 50 mol %. In the potential region around the PZC, processes that involve water are hydrogen oxidation and evolution reactions (HOR/HER), including the formation and desorption of H_UPD_ layers. As was shown in a previous paper,^33^ the H_UPD_ charge on Pt with [Dema][TfO] is poor and the extrapolated charge at a water content of zero is close to nil. This correlates with the high *R*_2_ values at low water contents close to the PZC. Moreover, it means that water molecules are the proton carriers and hydrogen adsorption proceeds *via* H_3_O^+^ instead of BH^+^, similar to the ORR.

It is known from the literature, that the conjugated base diethylmethylamine (the precursor of Dema^+^) has a poisoning effect in the H_UPD_ region, i.e., close to the potential of zero charge.^34^ This is not surprising, as at high negative or positive surface charges, the Pt surface is preferentially covered by either cations or anions and, if present, water dipoles with a favored orientation.^35^ Conversely, at potentials close to the PZC, there is a more diffuse distribution and random orientation of the ions and water molecules, respectively.^21^ This makes the Pt surface prone to poisoning by diethylmethylamine and would explain the maximum of *R*_2_ being close to the PZC as well.

## 4 Conclusions

In this study, we performed an impedance analysis of the electrochemical processes at the interface of a polycrystalline platinum electrode and a protic ionic liquid (PIL) electrolyte ([Dema][TfO]) as a function of the water concentration under oxygen saturation. To the best of our knowledge, it is the first impedance study of a platinum/PIL interface that considers both capacitive and Faradaic processes, including oxygen reduction reaction (ORR).

The following conclusions can be drawn:

- The analysis of the impedance spectra in the complex capacitance plane (CCP) using a Cole–Cole type equation similar to those that have been applied for (aprotic and) protic ionic liquids under nitrogen saturation is not appropriate for the same system under an oxygen atmosphere. This is not only because CCP plots are especially suitable for the analysis of capacitive rather than Faradaic processes but also as the resistances of the latter mask the midfrequency capacitive process, induce high fitting errors and so prevent the determination of reliable midfrequency capacitances.
- It is therefore essential to develop an equivalent circuit capable of describing both Faradaic and capacitive processes in the platinum/ionic liquid interface and that makes fitting errors as small as possible. Such an equivalent circuit was evaluated by comparing different equivalent circuits from physical and statistical viewpoints. Because of the complex (parallel/serial) connection of the elements in the equivalent circuit, an unambiguous correlation of the capacitance and CPE with only one of the two interfacial resistances is not possible.
- It is useful to study the influence of water on Faradaic and capacitive processes on platinum in protic ionic liquids over a wide range of water concentrations (here: almost neat ionic liquid to 80 mol % of water). This is because depending on the interfacial process, a significant change in the corresponding resistances and capacitances can occur at low or higher water contents.
- Whereas the larger interfacial resistance and capacitance/CPE can be well associated with Faradaic and capacitive processes, the nature of the small interface resistance remains unclear. However, it is probable that the small resistance correlates with fast interfacial processes, in which the electrode potential-dependent water and H_3_O^+^ concentration in the (innermost) double layer plays a decisive role.

To highlight the correlation between the structural properties of PILs and the ORR kinetics in greater detail, the investigation must be extended to a broader variety of different ionic liquids. This is of special importance for the future development of PEMFCs for operation in the temperature range of 100–200 °C.

## Supporting Information Available

Water content before and after EIS measurements; Bode plots of EIS measurements; comparison of complex capacitance plane plots under oxygen and nitrogen atmosphere; fitting results using different models, e.g., Randles circuit model, Cole–Cole type expression, CPE_1_–CPE_2_ model, and *C*_1_–CPE_2_ model (PDF)
