# Supplementary material for: Impedance Analysis of Capacitive and Faradaic Processes in the Pt/[Dema][TfO] Interface
Source: ACS Appl Mater Interfaces. 2024 Jan 22;16(4):5278–85. doi: 10.1021/acsami.3c15465 (PMC10835653; doi:10.1021/acsami.3c15465)
Supplement: Supplementary file 1 — am3c15465_si_001.pdf [file am3c15465_si_001.pdf]

Supporting information

Impedance Analysis of Capacitive and Faradaic Processes in the Pt/[Dema][TfO] Interface

Yingzhen Chen,[a,b] Klaus Wippermann, [a] Christian Rodenbücher,*[a] Yanpeng Suo,[a,b] and Carsten Korte [a,b]

[a] Institute of Energy and Climate Research – Electrochemical Process Engineering (IEK-14), Forschungszentrum Jülich GmbH, 52425 Jülich, Germany

[b] RWTH Aachen University, 52062 Aachen, Germany

*Email: c.rodenbuecher@fz-juelich.de

Contents

Water content before and after EIS measurements S2

Bode plots of EIS measurements S3

Comparison of complex capacitance plane plots under oxygen and nitrogen atmosphere S4

Fitting results using Randles circuit model S5

Fitting results using Cole–Cole type expression S6

Fitting results using CPE_1-CPE_2 model S7

Fitting results using C_1-CPE_2 model S8

Water content before and after EIS measurements

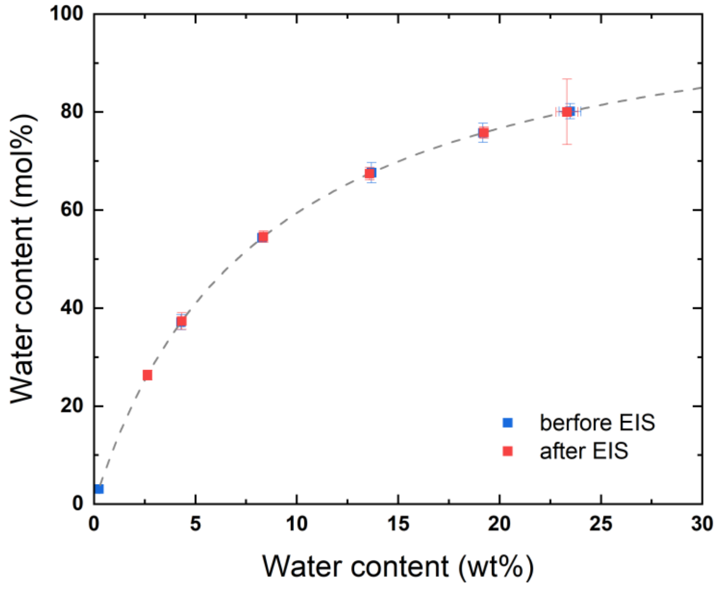

Figure S1 Comparison of the water content before and after EIS. Karl-Fischer titration was performed three times for each sample. The average values of the water content for the sample with the error bar are plotted.

Bode plots of EIS measurements

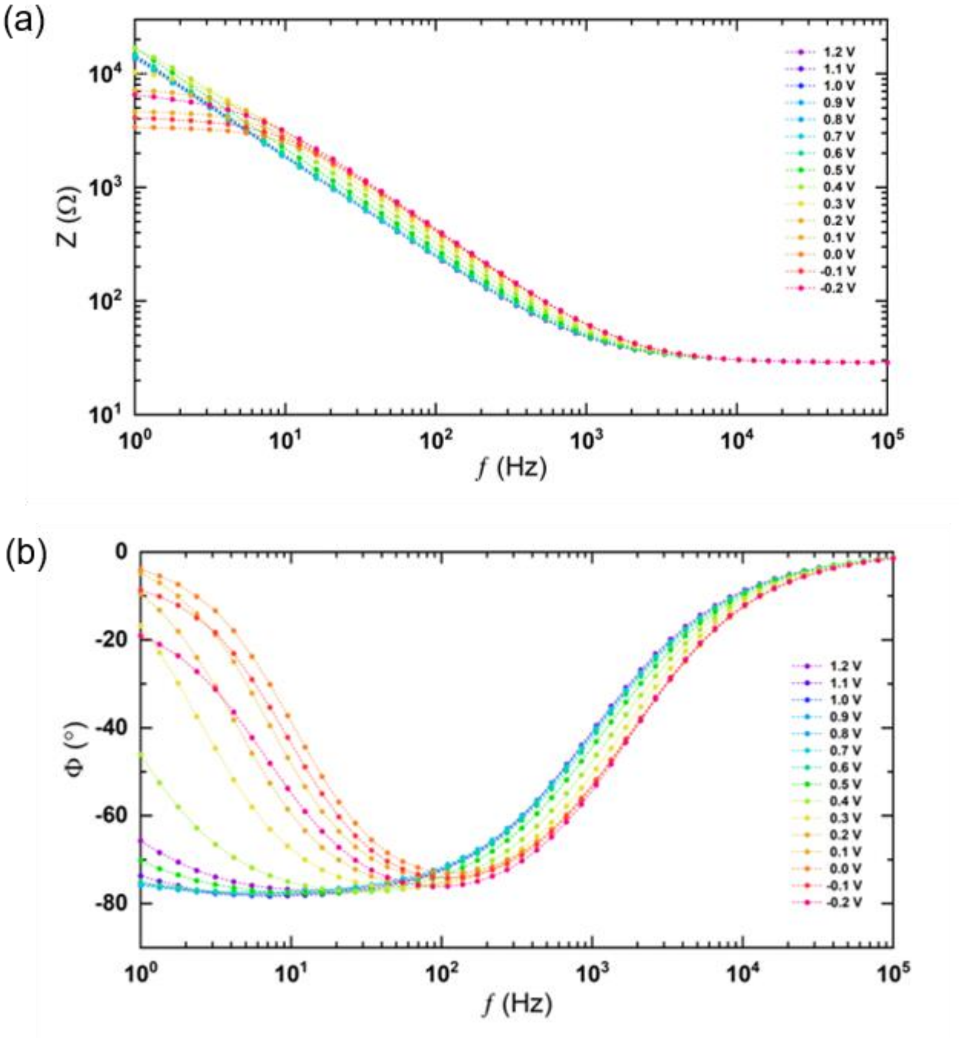

Figure S2 Potential-dependent impedance spectra of [Dema][TfO] with 50 mol% water at a Pt electrode under O_2 atmosphere represented by Bode plots; (a) impedance; (b) phase shift *vs.* frequency.

Comparison of complex capacitance plane plots under oxygen and nitrogen atmosphere

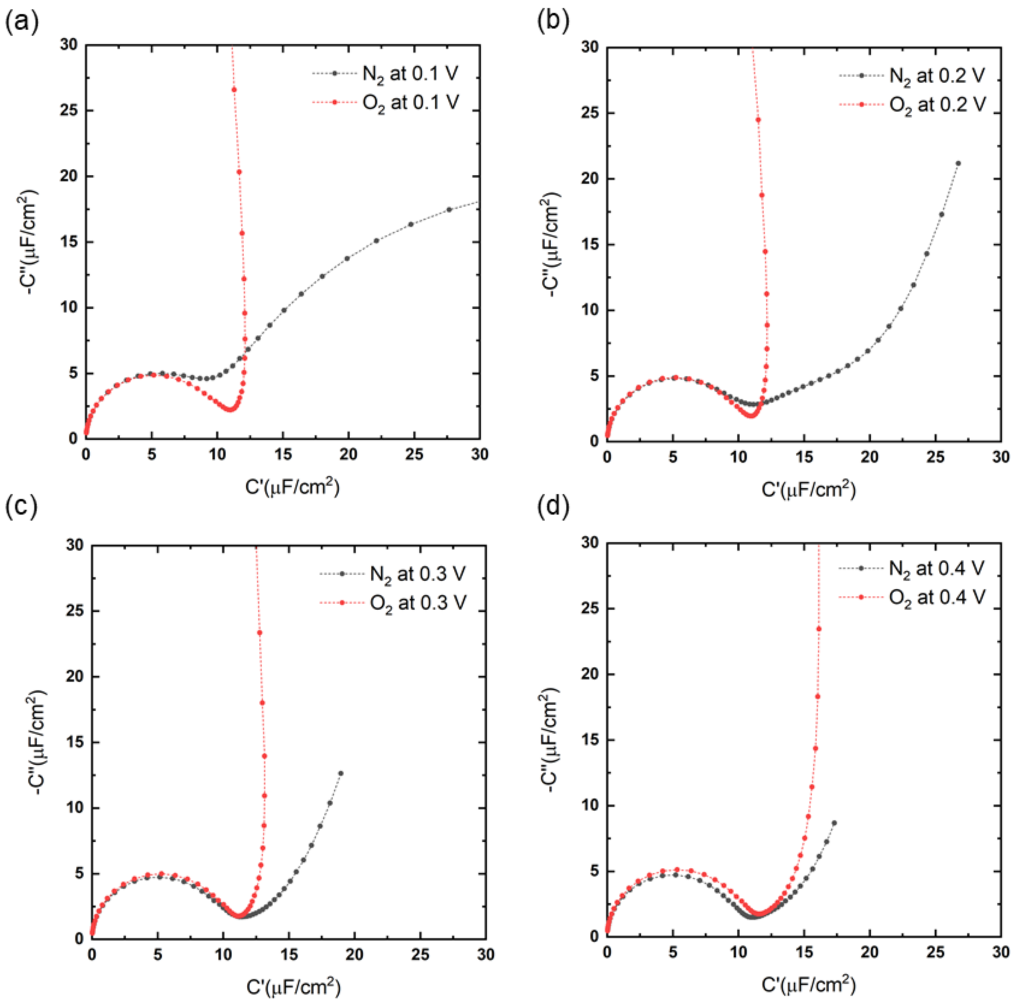

Figure S3 Complex capacitance plane plots of neat [Dema][TfO] at 90 °C under oxygen in the ORR region, *i.e.*, at potentials of (a) 0.1 V; (b) 0.2 V; (c) 0.3 V; and (d) 0.4 V, in comparison with that under a nitrogen atmosphere.

Fitting results using Randles circuit model

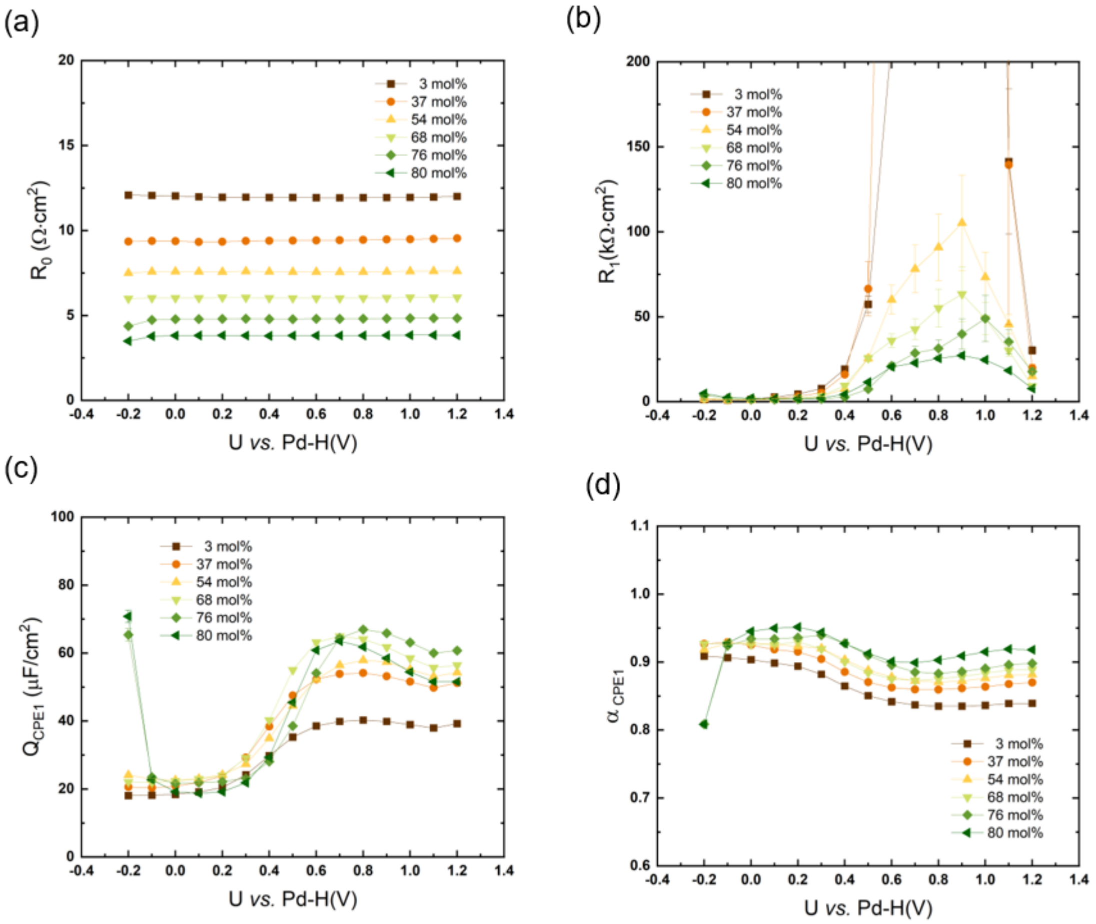

Figure S4 Fitted equivalent circuit parameters (a) R_0; (b) R_1; (c) Q_{CPE1} and (d) α_{CPE1} as a function of the potential using a Randles circuit model as shown in Figure 3(a).

Fitting results using Cole–Cole type expression

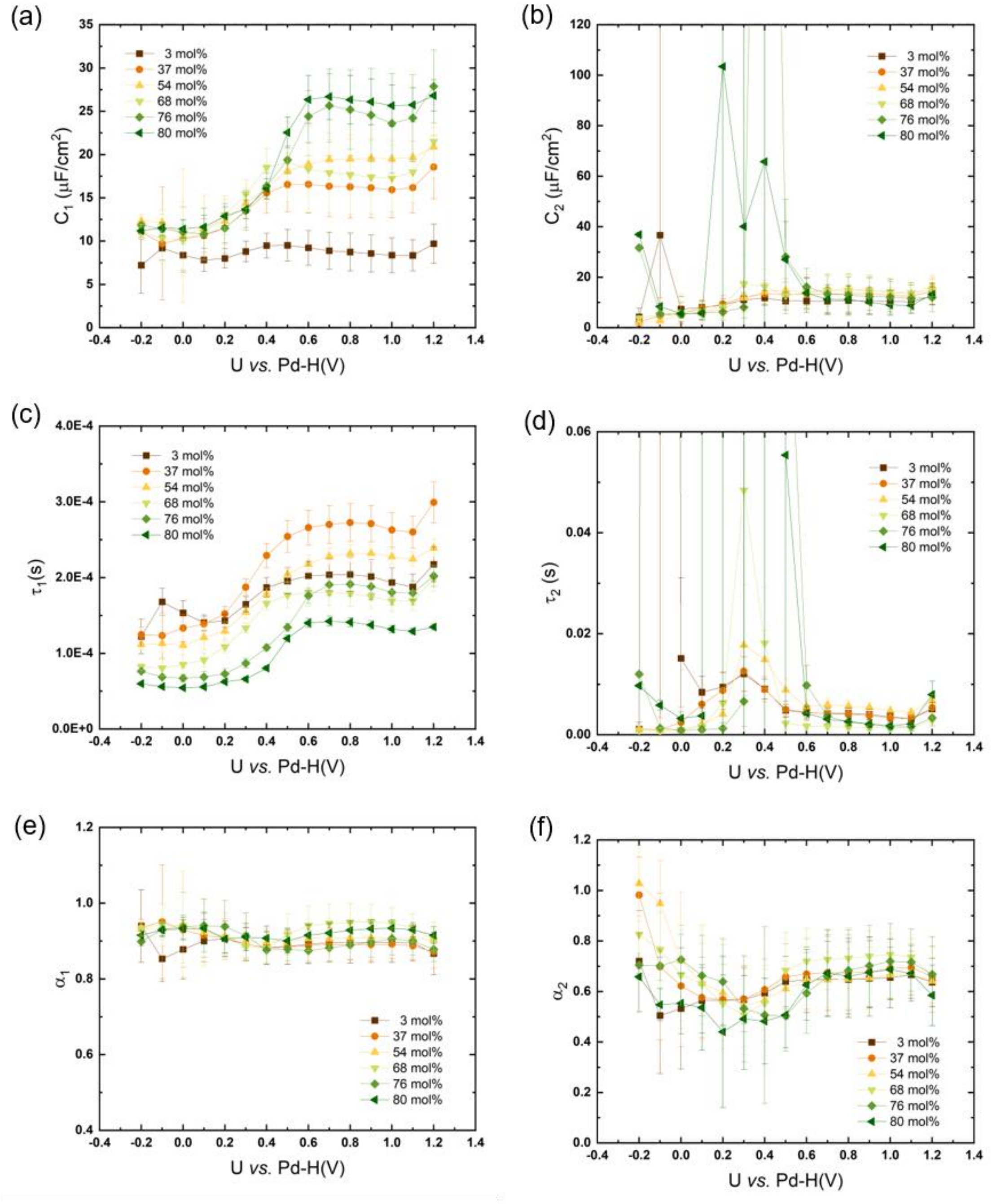

Figure S5 Fitted parameters (a) C_1; (b) C_2; (c) τ_1; (d) τ_2; (e)α_1 and (f) α_2 from the Cole–Cole type expression.

Fitting results using CPE_1-CPE_2 model

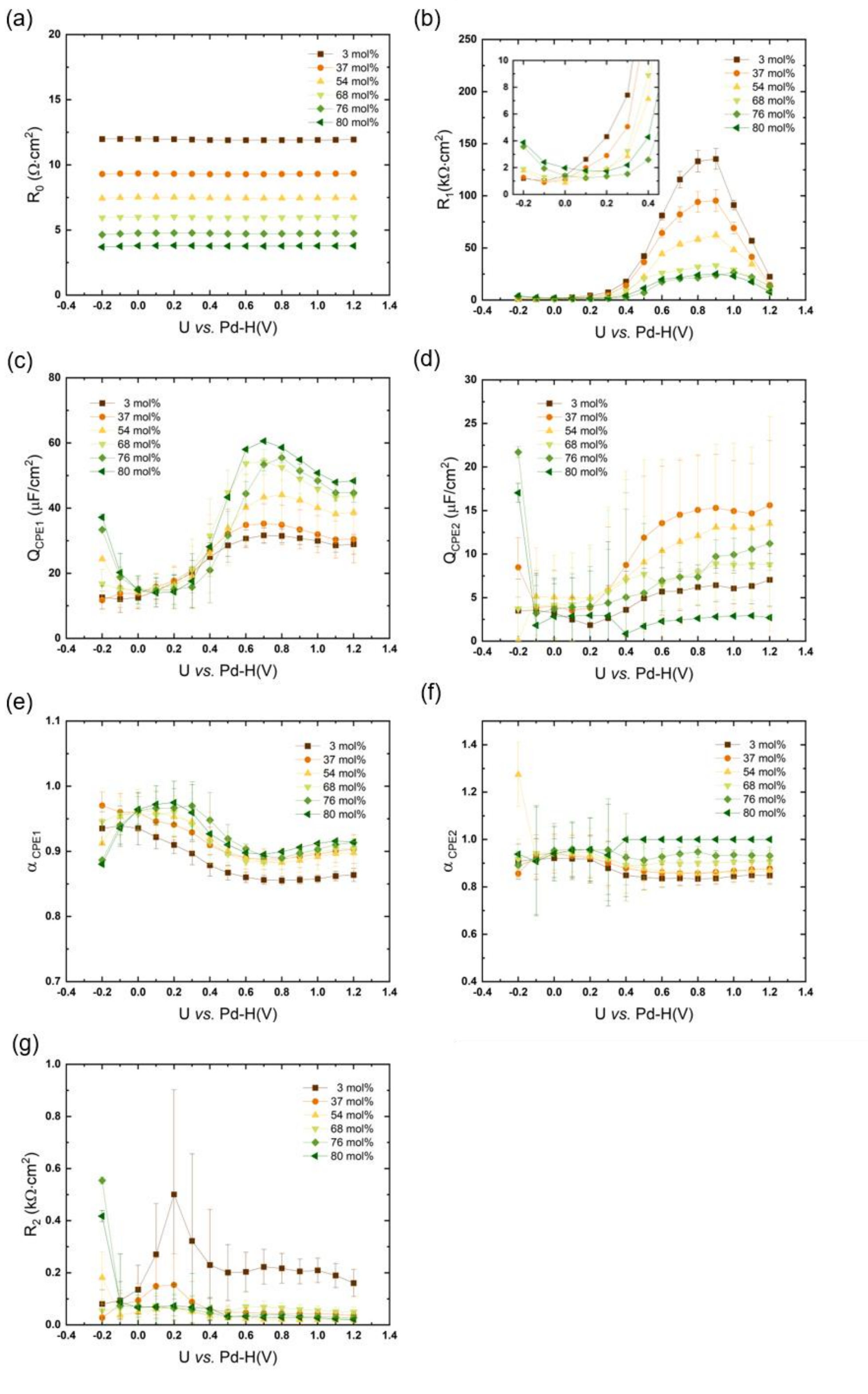

Figure S6 Fitted equivalent circuit parameters (a) R_0; (b) R_1; (c) Q_{CPE1}; (d) Q_{CPE2}; (d) α_{CPE1}; (e) α_{CPE2} and (f) R_2 as a function of potential using the model with CPE_1 and CPE_2, as shown in Figure 3(b).

Fitting results using C_1-CPE_2 model

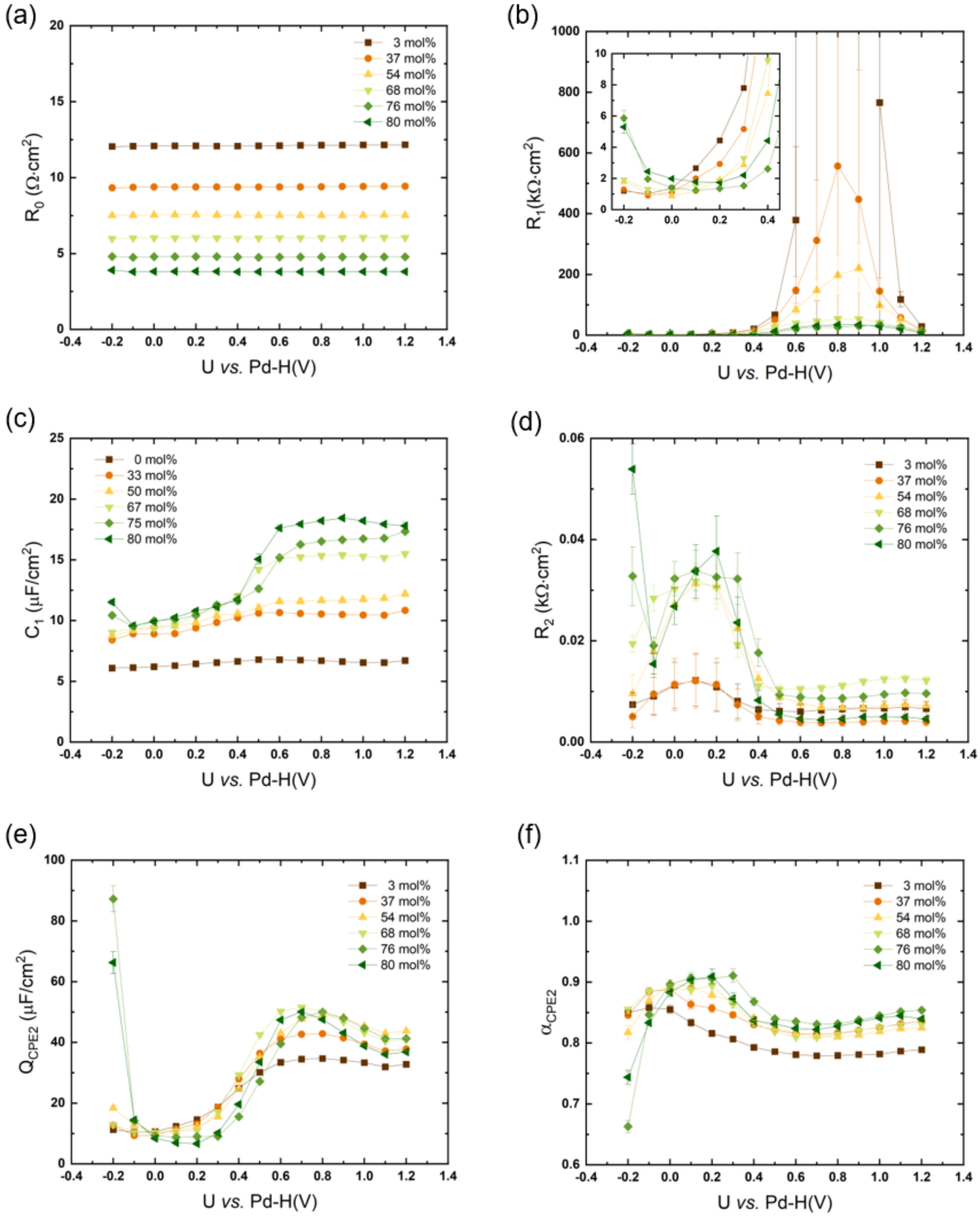

Figure S7 Fitted equivalent circuit parameters (a) R_0; (b) R_1; (c) C_2; (d) R_2; (e) Q_{CPE2} and (f) α_{CPE2} as a function of potential using the model with C_1 and CPE_2, as shown in Figure 3(c).
